# Serum proteome of the Egyptian rousette bat (*Rousettus aegyptiacus*) reveals signatures of immunity, proteostasis, and metabolism

**DOI:** 10.1038/s41598-026-46577-9

**Published:** 2026-04-06

**Authors:** Brooke N. Genovese, Nistara Randhawa, Benjamin A. Neely, Gabriela Grigorean, Amy J. Schuh, Brian R. Amman, Jessica A. Elbert, Simon J. Anthony, Jonna A. K. Mazet, Jonathan S. Towner, Brian H. Bird

**Affiliations:** 1 One Health Institute, School of Veterinary Medicine, University of California, Davis, Davis, CA USA; 2 Grand Challenges, University of California, Davis, Davis, CA USA; 3 National Institute for Standards and Technology (NIST), Charleston, SC USA; 4 Proteomics Core, Genome & Biomedical Sciences Facility, University of California, Davis, Davis, CA USA; 5 Viral Special Pathogens Branch, Centers for Disease Control and Prevention, Atlanta, GA USA; 6 Department of Pathology, College of Veterinary Medicine, University of Georgia, Athens, GA USA; 7 Department of Pathology, Microbiology, and Immunology, School of Veterinary Medicine, University of California, Davis, Davis, CA USA

**Keywords:** Chiroptera, Managed care, Non-model organism, Proteomics, Longevity, LCMS, Biochemistry, Computational biology and bioinformatics

## Abstract

Bats are increasingly studied for their ability to coexist with diverse viruses of human health importance. While this focus has yielded insights into host–pathogen dynamics, baseline physiological data from healthy bats remain limited, constraining comparative and mechanistic understanding. Serum proteomics offers a direct window into circulating proteins that underpin immune regulation, cellular maintenance, and metabolism. Here, we characterize the serum proteome of clinically healthy, captive Egyptian rousette bats (*Rousettus aegyptiacus*), the only known natural reservoir host species of Marburg virus. Using untargeted proteomic profiling, we identified and ranked over 400 proteins across major functional categories. Prominent findings included extensive representation of complement components spanning all activation pathways, high levels of interferon-responsive proteins, and abundant proteasome subunits, including immunoproteasome components. The dataset further revealed a robust profile of oxidoreductases and antioxidant enzymes, consistent with mechanisms of redox balance and iron regulation, alongside an apolipoprotein profile suggestive of dietary specialization. An unexpected finding was the unusually high abundance of type XX collagen, potentially linked to tissue remodeling demands of flight. Exploratory sex-based analyses suggested trends in stress response and immune-related proteins, although sample size limited statistical resolution. This work provides the first-look of the Egyptian rousette bat serum proteome, providing a reference point for cross-species comparisons and future studies of bat immunity, metabolism, and longevity. By identifying constitutive molecular features in healthy individuals, these findings expand the use of proteomics for understanding non-traditional model organisms and set the stage for future targeted functional investigations.

**Supplementary Information:**

The online version contains supplementary material available at 10.1038/s41598-026-46577-9.

## Introduction

Over the past decade, bats (of the order, Chiroptera) have captured growing scientific attention, not only for their ecological importance but also for their extraordinary biology: they live far longer than expected for their size^1^ and have a low incidence of cancer and other ageing-related diseases in spite of the high metabolic demands from flight^2,3^. Recent attention has been paid to certain species of bats that seemingly tolerate infections with microbes that are pathogenic in humans, including zoonotic coronaviruses, paramyxoviruses, and filoviruses, without showing clinical signs of disease^4–7^. Although understanding bats’ relationships with these microbes is of clear relevance to human health, the emphasis on bats as non-traditional models for human health investigations has largely overlooked baseline physiology in clinically healthy (i.e. no apparent signs of illness) and hindered the establishment of normative reference values for this important taxon. Compounding this issue, taxonomic-level investigations of bat biology are constrained by methodological and logistical hurdles, including limited availability of species-specific laboratory tools and reagents^8–10^.

Emerging proteomics approaches (alongside other -omic technologies) offer solutions to some of these technical barriers and open new possibilities for novel tools to study diverse species, including bats. Owing to their species-agnostic nature and minimal specimen requirements, proteomics is well-suited for studies that investigate preliminary host physiological states, host responses to different stimuli, and cross-species comparisons^11–17^. Despite growing interest in proteomics for non-traditional model organisms, baseline serum proteomics is underexplored in bats, especially frugivorous species.

Egyptian rousette bats (ERBs; *Rousettus aegyptiacus*), a frugivorous bat widely distributed across Africa and the Middle East^18^, are an ideal species for addressing the knowledge gap in the chiropteran proteome. ERBs are among the most well-studied species of bats^19–26^, but their research potential stems from their status as the natural reservoir host for Marburg virus, a filovirus that causes fatal disease in humans and other susceptible hosts (e.g. great apes)^27^. Yet, our understanding of the pathogenesis of this virus in its natural reservoir host is incomplete^28^. Ecological studies in ERBs describe seasonal virus shedding patterns linked to reproductive cycles^19^, but nutritional, reproductive, and/or anthropomorphic stressors may also influence shedding dynamics across a range of bat-associated microbes and in other frugivorous bat species^22,29–32^.

The extent to which ERBs exhibit distinctive proteomic adaptations reflecting their frugivorous diet, unique physiology, and extended longevity is unknown but proteomics is a valuable tool for building a deeper understanding of these complex host-pathogen dynamics in their natural host. Here, we address this gap by providing the first comprehensive serum proteomic characterization of clinically healthy captive-bred ERBs, establishing baseline protein abundance profiles within key functional categories related to research interests across bat physiology including immunity, proteostasis, metabolism, and redox regulation. These baseline datasets and analytic approaches will facilitate future studies to probe mechanisms of bat immunity, metabolism, and longevity, and to place bats more fully within comparative mammalian biology.

## Results

**Fig. 1:**
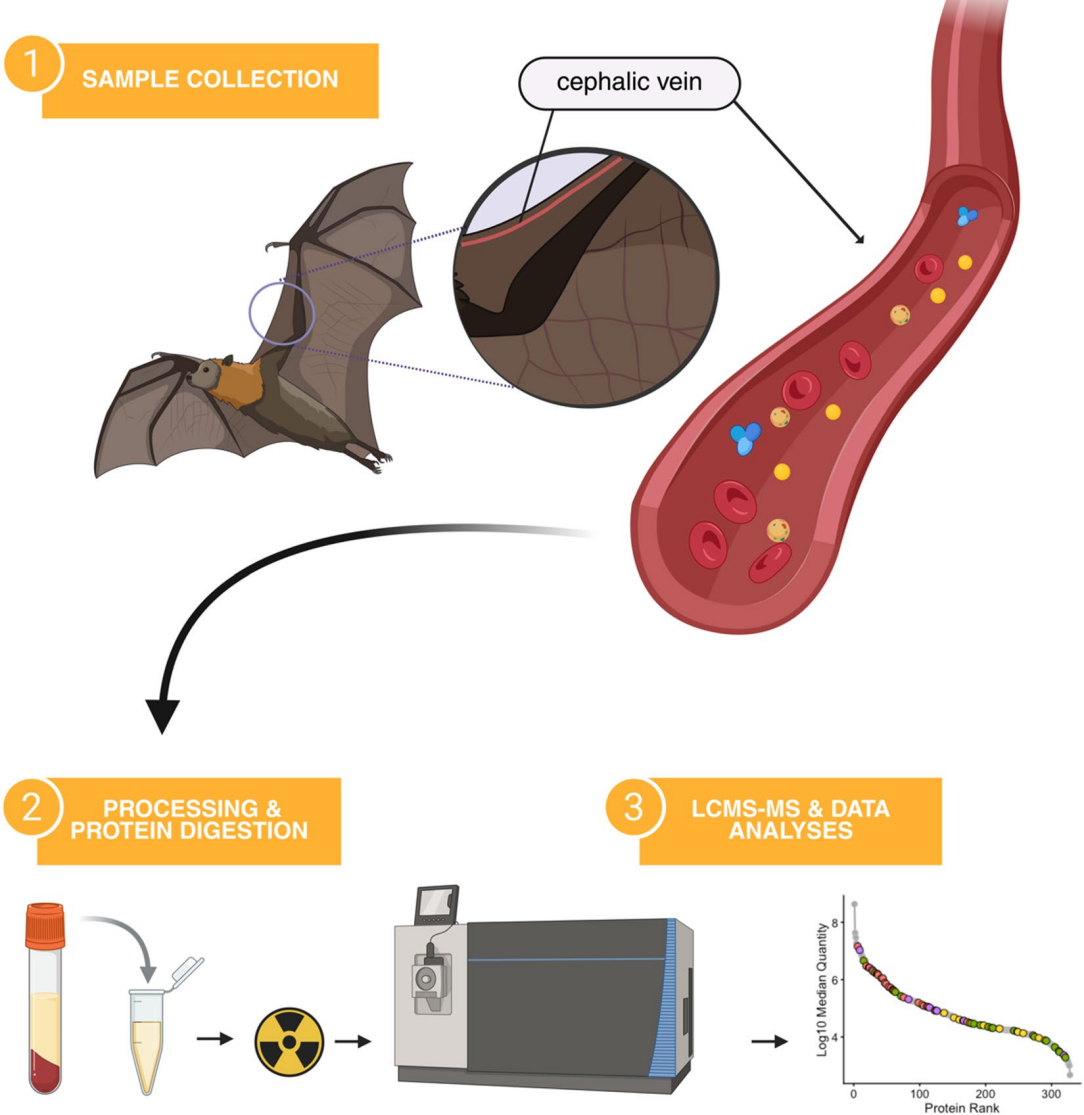
Overview of study design. Blood specimens were obtained from the cephalic vein of adult, captive-reared, healthy, non-pregnant Egyptian rousette bats (n = 6). The resulting serum samples were gamma-irradiated prior to performing protein digestion by the S-trap method (see ‘Methods’ for details). From here, the samples (peptides) were analyzed by “shotgun” liquid chromatography mass-spectrometry (LCMS/MS) operated in data-independent acquisition mode (DIA) and subsequently processed using Spectronaut 18. The resulting Spectronaut report, which included quantitative peptide and protein data, was the primary input for downstream bioinformatic analyses in R. Image created with Biorender.

### Characterization of the ERB serum proteome

**Fig. 2:**
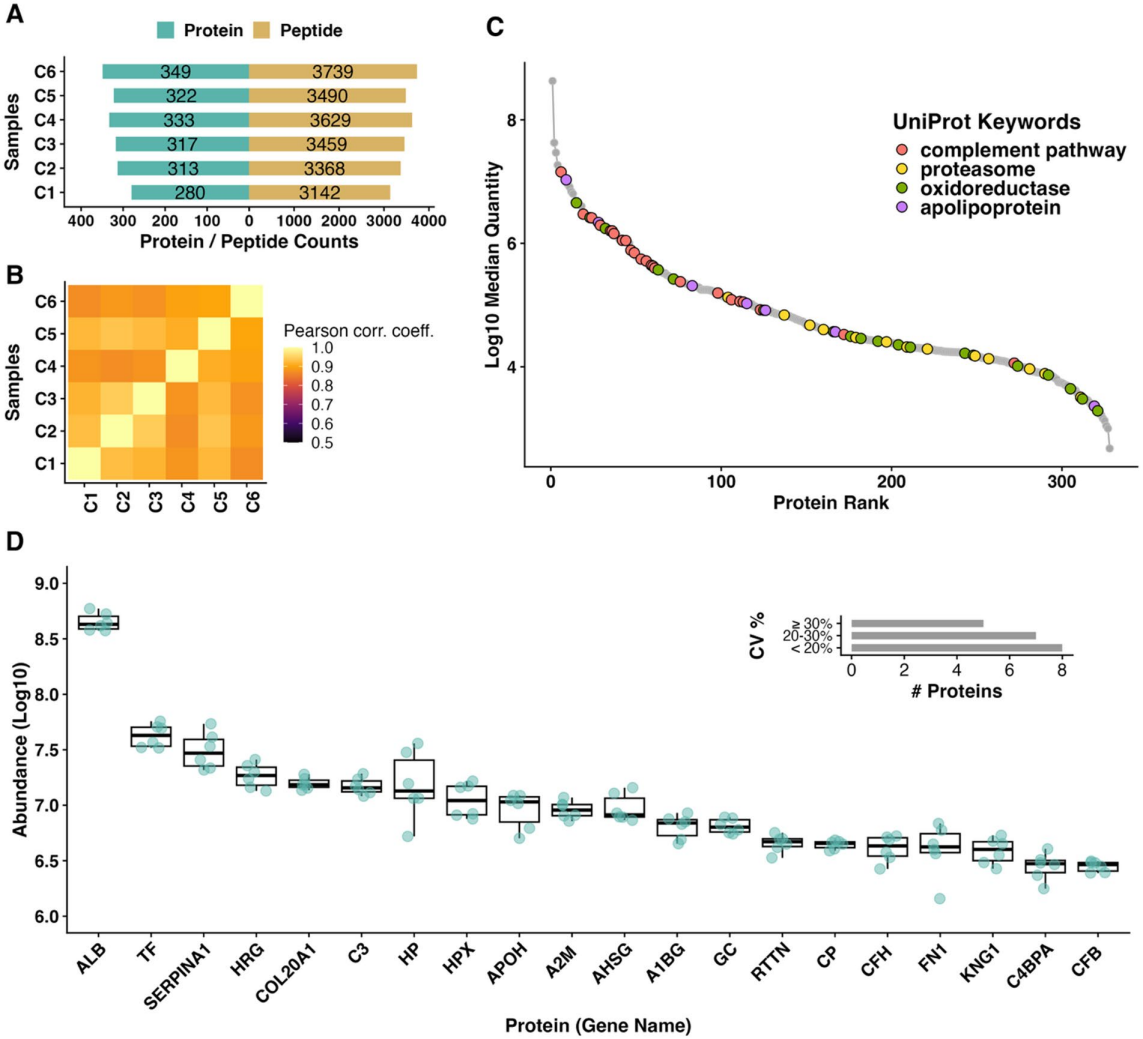
Characterization of clinically healthy, captive-reared ERB serum proteome. (**A**) Number of peptides and proteins detected per sample (post-data filtering and processing). Detected peptides have a confidence score (for identification) of < = 0.01 (S1). (**B**) Sample correlation heatmap. A correlation heatmap between samples with Pearson correlation coefficients (r) on a scale of 0.5 to 1.0. A correlation coefficient of 1 indicates a perfect positive linear correlation between sample pairs. Samples C1–C3 are from female bats and samples C4–C6 are from male bats. (**C**) Average protein abundance distribution by rank. All proteins are plotted as the average intensity (y-axis; log10 median quantity) and highest-to-lowest protein rank (x-axis). The colored dots represent different UniProt keywords (complement pathway, proteasome, oxidoreductase, apolipoprotein), showing these functional categories are distributed across the abundance range rather than clustering at specific abundance levels. (**D**) Distribution of the top 20 proteins with inset coefficient of variation (CV) histogram. The top 20 proteins are identified by their gene-symbol equivalent. Each cyan dot represents an individual sample measurement, showing the spread across the six samples. The inset histogram shows the CV distribution as an assessment of heterogeneity across samples. Most proteins have a CV of < 20%.

As an initial step towards defining baseline circulating protein profiles, we analyzed the blood serum proteome of age-matched, clinically healthy, captive-reared male and female ERBs (*n* = 3 male, *n* = 3 female; Fig. 1). This approach to serum protein profiling allowed us to perform quality control and statistical benchmarking across our uninfected dataset and to broadly characterize the relative abundance of both individual proteins and key protein classes. We quantified 401 proteins across these six specimens and retained 83% (332/401) of these proteins for abundance ranking (see Methods and supplemental for full list). The median number of quantified peptides and proteins in samples from uninfected bats were 3,474.5 ± 214.5 and 319.5 ± 22.2, respectively, with high correlation (*r* = 0.7–0.9) among sample protein profiles (Fig. 2A-B; S1; S7). We found that the serum proteome spans about 5 orders of magnitude in abundance and that albumin (ALB; log10 median quantity = 8.63) is the most abundant protein in serum (Fig. 2C-D). As expected, highly abundant serum proteins, including serotransferrin (TF) and haptoglobin (HP), were detected at ranks consistent with high abundance levels in mammalian circulation^15,33^.

Markers for hemolysis (such as HBB, FGA, FGB, or FGC) or other obvious technical artifacts were not detected among the top 20 most abundant proteins consistent with high quality specimen collection and pre-proteomic specimen handling (Fig. 2D). Given the lack of specific functional annotation for proteins in non-model organisms (such as bats) we used keywords that corresponded to each bat protein’s human ortholog (see “Methods” for details on the process for determining bat-human orthologs). Most identified proteins (88.7%) were annotated with keywords that were consistent with detection in circulating mammalian serum. The most frequent biological process representing 15.1% of serum proteins was “immunity” (KW-0391), which encompasses proteins that participate in an immune system process that functions in the response of an organism to a potential internal or invasive threat. Descriptions for these keyword annotations and their corresponding rank abundances are found in the supplemental materials (S2-S3).

#### Key protein categories

**Fig. 3:**
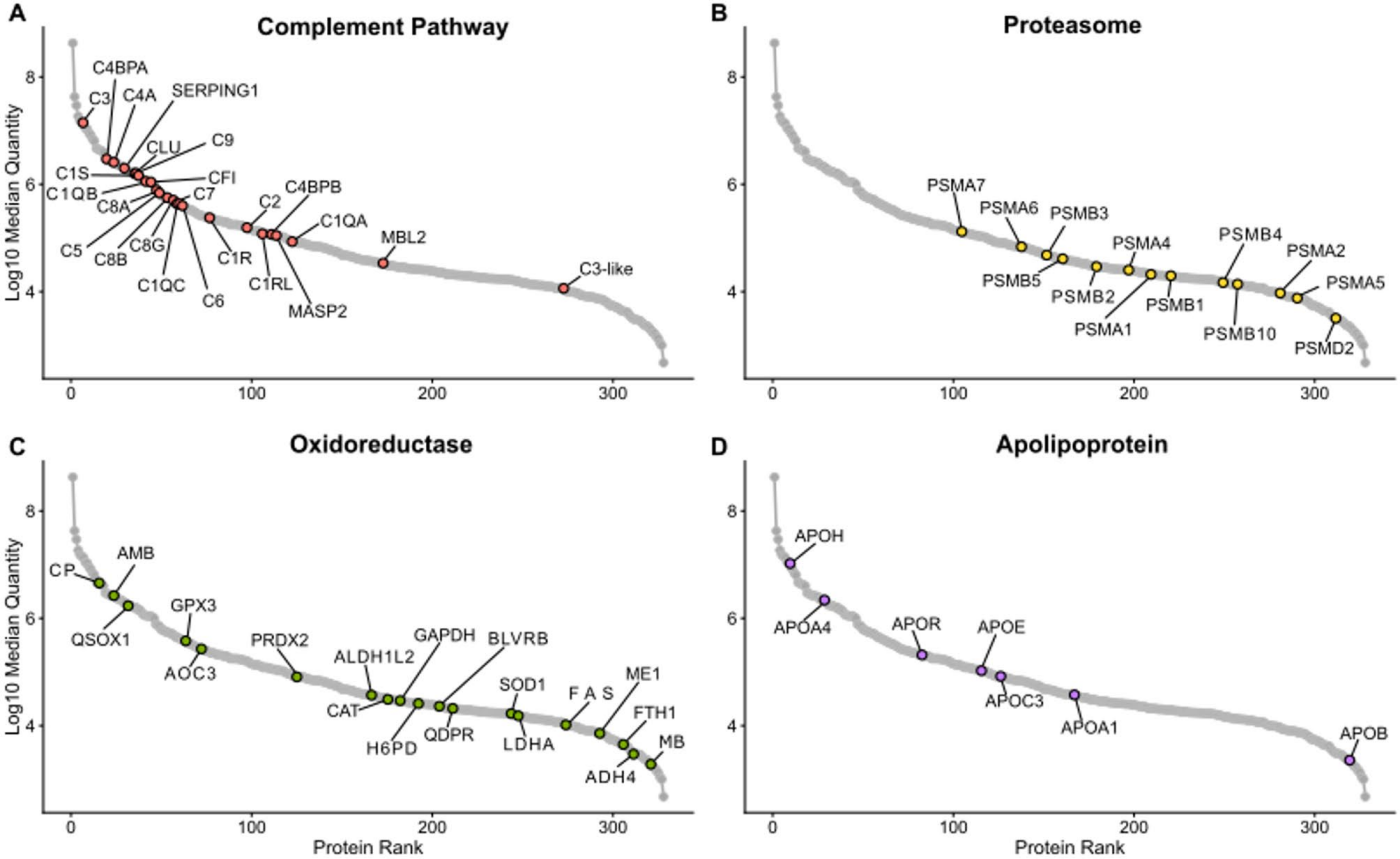
Rank abundance distribution of Egyptian rousette bat serum proteins by functional protein class. Proteins are plotted as the average intensity (y-axis; log10 median quantity) and highest-to-lowest protein rank (x-axis). The colored dots represent proteins that are annotated to respective UniProt keywords while all other proteins are shown in grey. Proteins are labeled with the human gene symbol for each bat protein. See Table 1 for complete protein names and ranking in serum.

To facilitate biological interpretation of the serum proteome, we categorized proteins into functional groups based on UniProt keyword identifiers. We selected four UniProt keyword identifiers related to protein categories of interest to bat physiology in the literature that characterize the circulating protein phenotype in clinically healthy, captive-reared ERBs, including: “complement pathway”, “proteasome”, “oxidoreductase”, and proteins categorized as apolipoproteins by conventional nomenclature (Figs. 2C and 3A-D). We encourage readers to explore the full list of protein rankings and their associated functional annotation in Supplemental Table 3.

#### Complement

The UniProt keyword (KW) terms describing proteins of the classical complement pathway (KW-0180), alternate complement pathway (KW-0179), and the lectin pathway (KW-1018) was used to categorize 24 serum proteins and determine their rank and abundance in the serum proteome (Fig. 3A). Complement proteins demonstrated consistently high abundance in ERBs, with 11 of the 24 detected complement components ranking within the top 100 most abundant serum proteins, and C3 ranking 6th overall (S8). Recognition and initiation components from both the classical pathway and lectin pathway (e.g. MBL2 and MASP2) were identified (Table 1). Proteins related to downstream cascade steps that are consistent with activation intermediates were also detected. Terminal pathway proteins included C5 - C9, corresponding to constituents of the membrane attack complex. Several regulators were also observed, including C4-binding protein subunits (C4BPA and C4BPB), clusterin (CLU), C1 inhibitor (SERPING1), and complement factor I (CFI). This broad coverage across initiation, amplification, and terminal phases indicates that the complement cascade machinery is well represented across functional categories in ERB serum, though functional activity would require experimental validation.

#### Proteosome

Repeating this strategy, our analysis also identified thirteen out of seventeen canonical proteasome subunits (KW-0647; Fig. 3B) constituting a near complete constitutive 20 S proteasome complex (Table 1). This included multiple α-core subunits (PSMA1, PSMA2, and PSMA4–7) as well as catalytic β-core subunits (PSMB1–5). Two canonical subunits, PSMB7 and PSMA3, were not detected. Among immunoproteasome components, only the inducible β subunit PSMB10 was observed, while PSMB8 and PSMB9 were absent. In addition, one regulatory 19 S subunit (PSMD2) was detected and indicative of circulating fragments of the 26 S proteasome complex.

#### Oxidoreductase

We next examined proteins with oxidoreductase-related activity (i.e. enzymes involved in the catalysis of an oxidation-reduction (redox) reaction) (KW-0560; Fig. 3C; Table 1). Several “classic” serum antioxidant enzymes were detected, including glutathione peroxidase 3 (GPX3), peroxiredoxin-2 (PRDX2), catalase (CAT), and superoxide dismutase 1 (SOD1) as well as known carrier proteins with oxidoreductase-like activity (e.g. CP, AMBP, MB). Additional extracellular detection included redox cofactors and proteins involved with electron transfer (e.g. QSOX1, AOC3, BLVRB). Most of the proteins in this category are known metabolic oxidoreductases, such as lactate dehydrogenase A (LDHA), malic enzyme 1 (ME1), fatty acid synthase (FASN), and hexose-6-phosphate dehydrogenase (H6PD), aldehyde dehydrogenase 1 family member L2 (ALDH1L2), alcohol dehydrogenase 4 (ADH4), and ferritin heavy chain (FTH1). This oxidoreductase profile portrays a complex redox environment maintained within bat serum that is explored in the Discussion.

#### Apolipoproteins

Among apolipoproteins, we detected APOA1, APOH, APOA4, APOB, APOR, APOC3, APOC4, and APOE (Fig. 3D). APOH was the most abundant apolipoprotein detected, ranking 9th overall in the serum proteome, while APOA4 also ranked relatively high at position 28. In contrast, APOB, a major structural component of low-density lipoprotein (LDL) particles, was among the least abundant proteins detected overall (rank #319). It should be noted that APOR was manually assigned due to the lack of a known human ortholog.

### Exploring sex-dimorphic protein abundance patterns in healthy ERB serum

Having characterized functional categories of proteins in ERB serum, we next examined whether protein abundance varied between the sexes. To identify sex-specific differences in protein abundance, we performed differential abundance analysis (DAA) between healthy female and male ERB serum proteomes. After applying filtering criteria to ensure high-confidence quantitation, 3,117 peptides corresponding to 272 proteins were retained for analysis. Given our limited sample size we targeted proteins with potential biological significance using dual criteria: adjusted p-values (e.g. q-values) of ≤ 0.05 and/or an absolute log fold change ≥ 1.50. While no proteins were statistically significant after false discovery rate (FDR correction), 15 proteins demonstrated a trend towards biologically meaningful differences in abundance between sexes (Table 1). The full output is available in the supplemental materials (S4) and corresponding data/code repositories.

We observed asymmetry in distribution of extreme differences in proteins (represented by z-scores) and found that detection differences were skewed towards male bats (S9), which also had a more extreme range of z-scores (z-score = 18.9 for myosin-9; A0A7J8JI27) compared to female bats (upper range z-score = −13.1 for myoglobin; A0A7J8JI11). As expected, proteins with known sex differences in mammals were among the most differentially abundant: for example, Mullerian-inhibiting factor (AMH) showed 8-fold higher abundance in male serum (log2FC = 3.0, z-score = 13.5), representing one of the most strongly sex-biased proteins in this dataset (Table 1).

We also assessed intra-sex variability in abundance of serum proteins by relative coefficient of variation (rCV) and included these results in the supplemental materials. The rCV comparison revealed that most proteins clustered along the identity line (rCV female ≈ rCV male), indicating broadly comparable within-sex variability for most quantitated proteins in these bats (S6, S10-S11). Some proteins showed putative sex-dependent heterogeneity in variation (e.g. LTF, PON1, S100A4), while others qualified as highly variable in both sexes (e.g. APOC3) or had consistent, “stable”, abundance levels across individuals of the same sex (e.g. C7, AGT, APOA1).

**Table 1:**
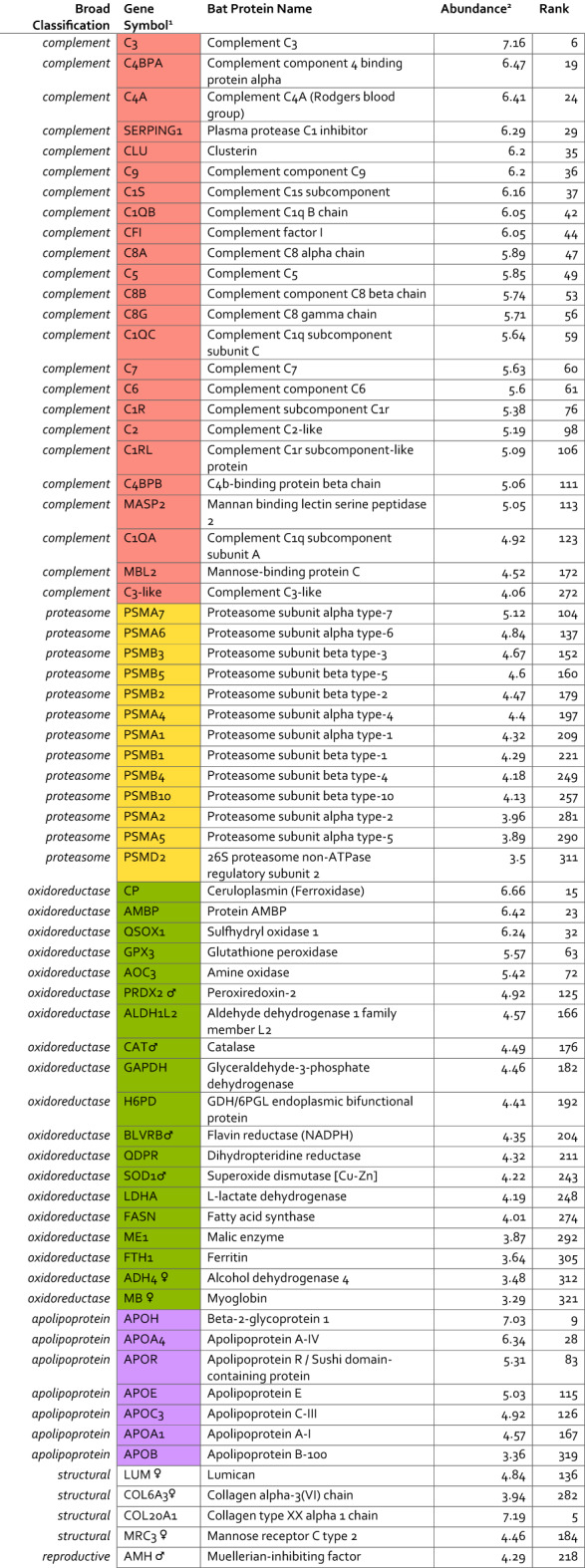
Rank abundance of serum proteins across major functional categories with representative sex-association.

^1^Symbols for male (♂) and female (♀) are used to denote proteins that tended to be higher in the indicated sex by either differential abundance or differential detection analyses. Results from these analyses are available in the supplementary. ^2^Abundance is represented as log10 median quantity.

## Discussion

Immunological research in bats has largely concentrated on their association with viruses and other microbes of high-consequence to human health^5,34^; thus, proteomic studies have focused on infection-related differences, with comparatively fewer studies examining the baseline proteome. Here, we map out the major functional categories of proteins circulating in clinically healthy captive-bred ERB serum that facilitate preliminary cross-species comparisons, as well as applied statistical approaches to uncover sex-based differences in abundance. While some findings were consistent with other species, such as high serum albumin, others revealed unexpected features across the ERB proteome that warrant deeper investigation.

### Serum proteomics reveals links among ERB immunity, proteostasis, and longevity

Given the interest in bats for their unique innate immune features and role in zoonotic disease transmission^34,35^, we identified and ranked by abundance proteins associated with the complement system. Though our study captures complement protein levels at a single timepoint in uninfected animals, rather than investigating infection dynamics, these baseline measurements offer insights into the constitutive immune landscape of healthy ERBs. In line with findings in other sub-populations of ERBs^36^ and other bat species^15^, complement components, like C3, were detected at high abundance in ERB serum. Not only did we observe C3 in the top 10 most abundant serum proteins in ERBs, but we also detected 24 complement-associated proteins spanning all three activation pathways described in humans and the membrane attack complex (Fig. 3A).

Beyond complement pathway components, several other proteins of the broader innate immune system were detected at relatively high abundance in ERB serum. Among these, SERPING1, an interferon-inducible inhibitor, ranked prominently (Fig. 3A). In humans, SERPING1 encodes the complement regulator C1–INH^37^, which is transcriptionally-induced by interferon gamma (IFN-γ) and elevated in multiple viral infections, including SARS-CoV-2^38^. Haptoglobin (Hp), a known acute-phase response protein, was also detected at relatively high levels in clinically healthy ERBs (Fig. 2D). This aligns with previous measurements in humans^33^, other mammals^15,39^, and in geographically-distinct sub-populations of ERBs^23^, where Hp increased markedly following experimental bacterial challenge but was also measurable at baseline. Although albumin and immunoglobulins (Igs) are typically among the most abundant serum proteins, the comparatively limited Ig representation here likely reflects database-related constraints inherent to discovery proteomics, as highly variable Ig sequences depend strongly on FASTA composition and curation; similarly, large human proteomics resources (e.g., the Human Protein Atlas) report relatively limited Ig identification despite their known biological abundance. Collectively, our results are consistent with these patterns while also highlighting potential inter-individual or population-level variability that could be influenced by environmental or handling factors.

Several studies of ERB immune responses to endemic viruses have proposed that these bats employ strategic complement activation that prevents self-damaging pathology^28,40^. In parallel, we detected the inducible immunoproteasome subunit PSMB10 and other IFN-responsive antigen-processing proteins, such as ERAP1, alongside the presence of type I IFN receptor subunits (IFNAR1/2)^41,42^. Although these proteins participate in distinct immune pathways, their co-detection points toward a serum proteome that retains multiple interferon-responsive elements and is consistent with findings from Bondet et al. (2021), who reported detectable circulating IFN-α protein in healthy ERBs^43^. The absence of a broader canonical ISG signature likely reflects either a steady-state immune profile in healthy animals or the inherent detection limits of untargeted serum proteomics for low-abundance cytokine-related proteins^44^. Future proteomic studies examining infection-challenged cohorts will be critical to determine how these proteins respond dynamically and to clarify their roles in antiviral and inflammatory processes.

Expanding our analysis beyond classical immunological proteins, we also assessed which proteasome subunits are detectable in the serum of healthy ERBs and detected 13 of 17 canonical proteasome subunits of the 20S proteasome (Fig. 3B; Table 1). Our findings present a strong parallel of both catalytic and structural components ranked relatively high in abundance in bat serum compared to humans^15,33^. Interestingly, while PSMB7 was not found in this study or previous serum proteomics work by Neely et al., we did detect the inducible immunoproteasome subunit PSMB10, which suggests active immune processes. In vertebrates, IFN-γ induces the synthesis of specialized immunosubunits (β1i, β2i, and β5i) that are cooperatively incorporated into nascent proteasomes, replacing their constitutive counterparts to form immunoproteasomes rather than constitutive proteasomes (20S)^45,46^. The 20S proteasome serves as a central component of cellular proteostasis networks in humans, responsible for degrading damaged proteins that arise from metabolic, environmental, and pathological stresses^47,48^. In human serum, circulating 20S proteasome concentrations have been reported in the range of ~ 0.2–2 µg/mL^38,48^, although these values are derived from absolute quantification approaches and are not directly comparable to the relative abundance metrics reported here.

While not a precise quantitative contrast across species, the relatively high levels of circulating 20S proteasome observed here (compared to humans and other mammals)^33^ raises intriguing questions about bat proteasomes and their role in responding to immunological challenges. Moreover, many theories surrounding bats’ remarkable longevity relative to their size center on the hypothesis that powered flight generates increased oxidative stress, requiring bats to evolve enhanced mechanisms for mitigating oxidative damage^49–52^. Recent comparative transcriptomic studies reveal that long-lived bats like *Myotis* species exhibit age-related compensatory increases in DNA damage signaling and repair pathways^53^, suggesting sophisticated cellular maintenance systems. Other studies on hibernating species of bats have found that they do not demonstrate loss of protein homeostasis or muscle mass during hibernation^54^. Given the proteasome’s central role in protein quality control and cellular homeostasis, future studies examining proteasome efficiency in vivo across bat lifespans could provide critical insights into how these animals maintain proteostasis, despite the oxidative challenges of their energetically intensive physiology.

Building on this theme of cellular maintenance, we next considered the suite of oxidoreductase-related proteins in ERB serum. In line with other studies of oxidative stress in ERBs^55^, we found that healthy ERBs exhibit a robust serum protein profile for redox management, with 19 oxidoreductases detected across diverse functional categories (Fig. 3C; Table 1). The most abundant oxidoreductases included ceruloplasmin (CP, rank #15), α−1-microglobulin/bikunin precursor (AMBP, rank #23), and quiescin sulfhydryl oxidase 1 (QSOX1, #rank 32), suggesting active copper transport, protease inhibition, and protein folding regulation, respectively^56^. Classical antioxidant defense enzymes were also observed, including glutathione peroxidase 3 (GPX3), peroxiredoxin-2 (PRDX2), catalase (CAT), and superoxide dismutase 1 (SOD1), indicating broad protection against reactive oxygen species^56,57^. We considered that these proteins can also be artefactually present in at high abundance in serum due to erythrocyte lysis or platelet contamination during specimen collection or sampling handling procedures^58^. Importantly for these analyses, our dataset does not support that these proteins are artefactually present, which would be evidenced by high-ranked fibrinogen chains, hemoglobin subcomponents, or other markers of questionable sample quality (e.g. hemolysis, specimen degradation) within our top ranked serum proteins (Fig. 2D). Moreover, previous studies by our group have investigated the impacts of gamma-irradiation on the serum proteome and have found little evidence that this inactivation technique would introduce oxidation products^59^.

The robust representation of iron-regulating oxidoreductases in ERB serum, particularly ceruloplasmin (CP, rank #15) and ferritin heavy chain, aligns with recent findings by Elbert et al. demonstrating that ERBs have a genetically determined affinity for iron accumulation^26^. Their study revealed that free-ranging ERBs naturally accumulate hepatic iron without associated morbidity, suggesting evolved mechanisms for managing high iron loads. Although not directly tested here the presence of GPX3 in our dataset may represent part of these protective mechanisms, potentially allowing ERBs to accumulate iron without suffering iron-mediated oxidative damage and contributing to the ferroptosis resistance observed in bat cells^52^. Thus, while ERBs possess robust iron scavenging systems, the expression and demand for these systems may vary between captive and wild populations, making it essential to consider dietary and environmental context when interpreting proteomic profiles for their physiological implications.

Given that frugivorous diets impose distinct metabolic demands^60^, we investigated whether ERBs show corresponding adaptations in their circulating apolipoprotein profile. In ERBs, we detected a broad panel of apolipoproteins (Fig. 3D; Table 1) although several APOs reported in other bat species (e.g. APOA2, APOC1, APOM)^15,61^ were absent. We found that APOB ranked among the lowest-abundance serum proteins (#319), in sharp contrast to its prominence in sanguivorous and insectivorous bats^15,62^ and in other mammals where it is a dominant lipoprotein component, often ranking within the top 20 serum proteins^17,39^. This might reflect dietary specialization: a frugivorous diet low in fat and cholesterol may diminish the need for APOB-containing lipoproteins (chylomicrons, VLDL, LDL)^60^. In contrast, the relative prominence of APOA1, APOA4, and APOE points to an emphasis on HDL- and triglyceride-related pathways^63^, potentially representing a frugivory-associated specialization in lipid metabolism. Despite this streamlined APO profile, APOC3 was retained in our dataset. In humans, APOC3 is a potent inhibitor of triglyceride clearance and has well-established links to inflammatory signaling, highlighting its dual role at the interface of metabolism and immunity^64^. Its presence in ERBs suggests that elements of triglyceride regulation and associated immune pathways are maintained, even in a frugivorous species. This interpretation, in which certain APOs related to LDL are not enriched in ERBs, aligns with experimental work in Jamaican fruit bats showing that dietary fat composition alters systemic metabolism and viral shedding dynamics^31^; thus, while preliminary, these findings present an interesting perspective into the tight links between diet, lipid handling, and immune function in fruit bats.

Beyond the major functional categories discussed above, we observed a notable finding that warrants separate consideration: COL20A1 (collagen type XX alpha 1) ranked as the 5th most abundant protein in ERB serum (Fig. 2D). This finding does not align with the complement, proteasome, oxidoreductase, or apolipoprotein pathways we have emphasized, yet represents a striking departure from typical mammalian serum proteomes, in which collagens are generally present at much lower concentrations. High circulating collagen levels typically indicate active extracellular matrix turnover and tissue remodeling processes^65^. In the context of fruit bats, this finding may reflect the unique mechanical demands of flight, ongoing wing membrane maintenance, or species-specific adaptations related to their specialized anatomy^3^. While the functional significance of elevated COL20A1 in healthy bat serum requires further investigation, this finding suggests that structural tissue maintenance represents a significant physiological priority in ERBs, potentially distinguishing them from terrestrial mammals with less dynamic structural demands.

### Preliminary evidence of sex-specific proteomic signatures

Given evidence of sex-based immune and metabolic variation in other mammals^57^, we investigated whether similar patterns were detectable in ERB serum proteomes. Male ERBs showed elevated trends in proteins related to cellular machinery, regulatory processes, cytoskeletal modeling, and stress response (Table 1) compared to females, but these results were not found to be statistically significant. However, the magnitude of fold-change among some proteins was striking, for example the metabolic enzyme BLVRB was nearly 5-fold higher in serum abundance in males and antioxidant enzyme levels from PRDX2, CAT, and SOD1 collectively amounted to be 4-to-7-fold higher in serum abundance relative to female bats. In contrast, our results show a more limited set of elevated proteins in female ERBs that are functionally related to tissue structure and maintenance (LUM)^66^ and immune surveillance (MRC3). Detection-based analysis also suggested sex-specific differences in innate immune proteins, such as CD109 antigen (CD109; 2-fold higher in females).

Even though our sample size limits strong conclusions, these results provide preliminary evidence of sex-specific proteomic trends in ERB serum, with males exhibiting higher abundance of proteins involved with managing cellular stress over females. Although this pattern contrasts with the general mammalian trend, in which males typically experience higher oxidative stress and females greater antioxidant protection^67,68^ it may reflect more efficient mitochondrial function in females, leading to lower ROS production despite lower baseline antioxidant levels^69^. Our findings suggest that sex influences serum proteomic landscapes in bats in ways that may diverge from established mammalian norms, opening avenues for future research and reinforcing the need for sex-balanced study designs in proteomic investigations.

## Conclusions

This comprehensive proteomic characterization of ERB serum represents a first look at the molecular landscape of an important bat species at the One Health interface. The depth of insights revealed through this analysis span complement pathways, protein homeostasis, metabolism, and oxidative stress, demonstrating the complexity of circulating proteins that underlie bat physiology. Moving forward, this dataset will provide a reference point and enable more targeted investigations into mechanisms underlying bat longevity, immunity, and viral coexistence – research questions that are fundamental to both comparative biology and public health preparedness.

## Methods

### Bats and biosafety

The captive-born bats used in this study originated from an established ERB breeding colony at the Centers for Disease Control and Prevention (CDC, Atlanta, GA, USA)^70^. Work with ERBs complies with and was approved by the Institutional Animal Care and Use Committee (IACUC) of the CDC, Animal Care and Use Program Office (ACUPO), Comparative Medicine Branch (CMB) and Institutional Biosecurity Board (IBB), using guidelines established by the Association for the Assessment and Accreditation of Laboratory Animal Care, International (AAALAC), the Animal Welfare Act and Regulations, The Guide for the Care & Use of Laboratory Animals, and was conducted following the relevant ARRIVE guidelines. The CDC is accredited by the Association for Assessment and Accreditation of Laboratory Animal Care International (AAALAC)^28,70,71^. While in managed care, the bats are fed a variety of chopped fruits (banana, cantaloupe, grapes, apples, pears) supplemented with Lubee Fruit Bat Supplement (HMS Zoo Diets Inc) and fruit juice, and are provided water ad libitum. All investigators and animal handlers followed strict BSL-3 safety and infection control practices.

Bats used in this study were selected based on availability of immunologically naïve (e.g. unexposed) animals and the constraints of BSL-3 laboratory space. All female bats were reproductively mature but confirmed not pregnant at the time of specimen collection. The bat groups were age- and sex-matched adults and no other methods of randomization were used in the selection of animals for this study.

### Specimen collection and inactivation procedures

Blood samples were collected by venipuncture of the cephalic vein on the propatagium using a sterile blood lancet (Premiere #95–7820, C&A Scientific, Manassas, VA, USA). Serum was separated from whole blood by centrifugation at 12,000 RCF for 90 s prior to inactivation procedures. All specimens used in this study received 5 megarads of gamma-irradiation while on dry ice^59,72^.

### Serum specimen preparation

Samples assigned an alphanumeric key and allocated into random batches for digestion and instrumentation procedures. For the digestions, all inactivated serum specimens were thawed to room temperature on wet ice and spun down and then 5 µl of each serum sample was aliquoted into prepped Eppendorf tubes containing 5% sodium dodecyl sulfate (SDS; volumetric ratio), 50 mmol/L triethylammonium bicarbonate (TEAB) buffer, and LCMS-grade water. Proteins were digested via suspension-trap devices (S-Trap; ProtiFi, 100 µg to 300 µg binding capacity). The S-Trap is a powerful Filter-Aided sample preparation (FASP) method that traps acid aggregated proteins in a quartz filter prior to enzymatic proteolysis and allows for reduction/alkylation/tryptic proteolysis all in one vessel. The enzymatic digestion was initiated with a first addition of trypsin 1:100 enzyme: protein (mass fraction) for 4 h at 37 °C, followed by a boost addition of trypsin using same wt/wt ratios for overnight digestion at 37 °C. Peptides were eluted from the S-Trap with sequential elution buffers of 100 mmol/L TEAB, 0.5% formic acid, and 50% acetonitrile 0.1% formic acid (all volumetric fraction). The eluted tryptic peptides were dried in a vacuum centrifuge and re-constituted in 0.1% trifluoroacetic acid (volumetric fraction). These were subjected to liquid chromatography mass spectrometry (LCMS) analysis.

### Liquid chromatography-mass spectrometry (LCMS) of serum samples

Peptides were resolved on a Thermo Scientific Dionex UltiMate 3000 RSLC system: PepSep 150 μm x 25 cm C18 column (PepSep; Denmark) with 1.5 μm particle size (100 Å pores), heated to 40 °C. A sample volume of 5 µL was injected corresponding to 1 µg of total peptide, and separation was performed in a total run time of 90 min with mobile phases A: water, 0.1% formic acid, and B: 80% acetonitrile, 0.1% formic acid (volumetric fractions). Separated peptides were electrosprayed directly into a tribrid Lumos mass spectrometer (Thermo Fisher Scientific), operated in data-independent acquisition mode (DIA). A survey full scan MS (from m/z 350 to 1200) was acquired in the Orbitrap at a resolution of 120 000 (at 200 m/z). For the fragmentation spectra, the following settings were used: the mass range of 350–1200 Da was segmented into 19 windows, overlapping by 1 Da, with an isolation window width of 45.7 Da. They were fragmented via higher collisionally-induced dissociation (HCD) at 33% normalized collision energy. AGC target was set to 1000% (5 × 10^5^) and ion filling time was set to automatic. Detection was in the Orbitrap at a resolution of 15 000.

### Data processing with Spectronaut

LC-MS files were processed with Spectronaut version 18 (Biognosys, Zurich, Switzerland) using DirectDIA analysis mode. Mass tolerance/accuracy for precursor and fragment identification was set to default settings. A maximum of two missing cleavages were allowed, the required minimum peptide sequence length was 7 amino acids, and the peptide mass was limited to a maximum of 4600 Da. Carbamidomethylation of cysteine residues was set as a fixed modification, and methionine oxidation and acetylation of protein N termini as variable modifications. A decoy false discovery rate (FDR) at less than 1% for peptide spectrum matches and protein group identifications was used for spectra filtering (Spectronaut default). The FDR of 1% was set at the peptide spectrum match (PSM) level, as well as at 1% at protein level. For downstream analysis, a report was generated and exported from Spectronaut using the scheme provided by Mass Spectrometry Downstream Analysis Pipeline (MS-DAP; version 1.0.6; which is available at https://github.com/ftwkoopmans/msdap*)*^73^. The quantitative report exported from Spectronaut was processed using the MS-DAP R package, which was also used for preprocessing and quality control of this dataset prior to differential expression/abundance analysis.

### Bioinformatic analyses & protein ortholog mapping

All downstream bioinformatic analyses were performed using R version 4.4.1 and a suite of packages from *tidyverse*^74^, *proteomicsCV*^75^, and *MS-DAP*. Plots were generated using *ggplot2*^76^.

#### Protein sequence retrieval

Bat protein identifiers were obtained from DIA proteomic analysis results generated via Spectonaut and MS-DAP. The FASTA file used for searching bat samples corresponds to proteome ID UP000593571. The leading protein accession for each entry was extracted from concatenated protein ID strings. Unique accession numbers were compiled and used to retrieve corresponding protein sequences in FASTA format from the UniProt Knowledgebase using the UniprotR package^77^ (v2.3.0). The GETSeqFastaUniprot() function was used to download FASTA sequences for all unique bat protein accessions.

#### Reference human protein database preparation

The reviewed Swiss-Prot human proteome was obtained from UniProt via API (09–2024). Protein sequences were downloaded in FASTA format using a query restricting entries to Homo sapiens (taxonomy ID: 9606) and reviewed status. The resulting file was converted into a BLAST-compatible database using NCBI BLAST+ specifying the database type as ‘protein’.

#### Sequence similarity search (BLASTp)

To identify human orthologs of bat proteins, pairwise sequence similarity searches were conducted using BLASTp implemented via the *rBLAST* package. Bat protein sequences in FASTA format were read using the R package *Biostrings*^78^ and a BLAST database object was generated for the human refence proteome. We then performed BLASTp searches of all bat proteins against the human reference database and obtained alignment results for each query sequence.

#### Top hit determination and ortholog assignment

To identify the best match for human orthologs to bat proteins, BLASTp results were ranked according to alignment quality metrics. For each query sequence, hits were first sorted by ascending e-value, then in descending order of bitscore, and finally by descending order of percent identity (pident) in case of ties. A custom R function was written to extract the top ranking hit for each unique query protein based on this sorting hierarchy. A master results table was constructed by merging statistical outputs from MS-DAP with the bat-human ortholog top hits obtained via BLASTp similarity searches. As a final quality control step, the merged datasets were screened for unmatched entries (i.e. any bat proteins that did not “match” to a human ortholog protein). Since ortholog mapping to human proteins only works if the human ortholog is present, the results table was manually inspected for unmatched entries as well as known mis-matched proteins and modified accordingly. Manual adjustments are noted in the supplemental (S3).

### Key protein categories

To facilitate biological interpretation of the serum proteome, we categorized proteins into four functional groups. Categories were determined a priori based on recurring themes in the bat immunology and physiology literature and to span multiple facets of research interest in this system. Specifically, UniProt keyword identifiers were used to define proteins annotated to the complement pathway, proteasome, and oxidoreductases, while proteins conventionally designated as apolipoproteins were grouped according to established nomenclature. These categories were then used to examine rank abundance distributions and to highlight pathways of interest. This approach provided a consistent and biologically-interpretable structure aligned with the aims and scale of the present dataset.

### Differential abundance and detection analyses

Differential abundance (DAA) and detection analyses (DDA) were performed using MS-DAP. Differential detection analysis was conducted in parallel with DAA to capture proteins that may be missed by traditional expression-based approaches. We included the *DEqMS* algorithm, which accounts for variance dependence on the number of quantified peptides (i.e. PSM counts) per protein, using log-transformed and normalized protein abundance values^79^. To maximize reliable features for quantification in our pairwise analysis of these two groups of samples (female vs. male), only peptides with a sufficient confidence score in at least 2 samples per group were used for quantitative analysis. Common contaminants (e.g. human keratins, detergents) were also excluded from further analysis. The Variance Stabilizing Normalization (VSN) and mode between protein algorithms were applied sequentially and used for normalization. Filtering settings were set such that the minimum number of peptides per protein was set to 1 (after applying above filtering). Statistical contrasts were described by ‘group’, where values for group were either ‘female’ or ‘male’. Our analysis configures a series of linear regression models for protein measurements per statistical contrast (i.e. a specific hypothesis test comparing the means between each sex) adjusting for any batch effects. Less stringent criteria for DDA meant that peptides with a sufficient confidence score in at least 1 sample per group were used for quantitative analysis. For differential detection (DD), where we wanted to detect proteins with representation in one sex but not necessarily the other, we allowed proteins with 1 peptide if they were observed in at least 2 samples per sex (e.g. ‘group’).

## Supplementary Information

Below is the link to the electronic supplementary material.

Supplementary Material 1

Supplementary Material 2

## Data Availability

The mass spectrometry proteomics data have been deposited to the ProteomeXchange Consortium via the PRIDE partner repository with the dataset identifier PXD072134. The full code for these analyses is publicly available at GitHub at https://github.com/brookegenovese/baseline-bat-proteomics.
