# Supplementary material for: Serum proteome of the Egyptian rousette bat (Rousettus aegyptiacus) reveals signatures of immunity, proteostasis, and metabolism
Source: Sci Rep. 2026 Apr 6;16:16532. doi: 10.1038/s41598-026-46577-9 (PMC13216534; doi:10.1038/s41598-026-46577-9)
Supplement: Supplementary file 2 — Supplementary Material 2 [file 41598_2026_46577_MOESM2_ESM.docx]

**Serum proteome of the Egyptian rousette bat (*Rousettus aegyptiacus)* reveals signatures of immunity, proteostasis, and metabolism**

Genovese, B.N., Randhawa, N., Neely, B.A., Grigorean, G., Schuh, A.J., Amman, B.R., Elbert, J.A., Anthony, S.J., Mazet, J.A.K., Towner, J.S., and Bird, B.H*.

**Supplementary Excel File: SciRep_supplemental.xlsx**

- S1 (meta) = Metadata describing ERB specimens (n=6).
- S2 (rank) = Protein rank abundance and ortholog matching.
- S3 (keywords) = UniProt Keyword annotations.
- S4 (DAA) = Differential abundance analysis (DAA) results between female and male bats.
- S5 (DD) = Differential detection analysis (DDA) results between female and male bats.
- S6 (rCV)= Relative coefficient of variation (rCV) calculations.

**Supplementary PDF File: SciRep_supplemental.pdf**

- S7: Principal Component Analysis (PCA)
- S8: Abundance distribution of complement pathway proteins relative to the global proteome
- S9: Differential detection histogram (female vs male)
- S10: Coefficient of variation (rCV) comparison between sexes for proteins quantified in both male and female ERB serum
- S11: Methods for protein variability analysis

**S7: Principal Component Analysis (PCA)**

**
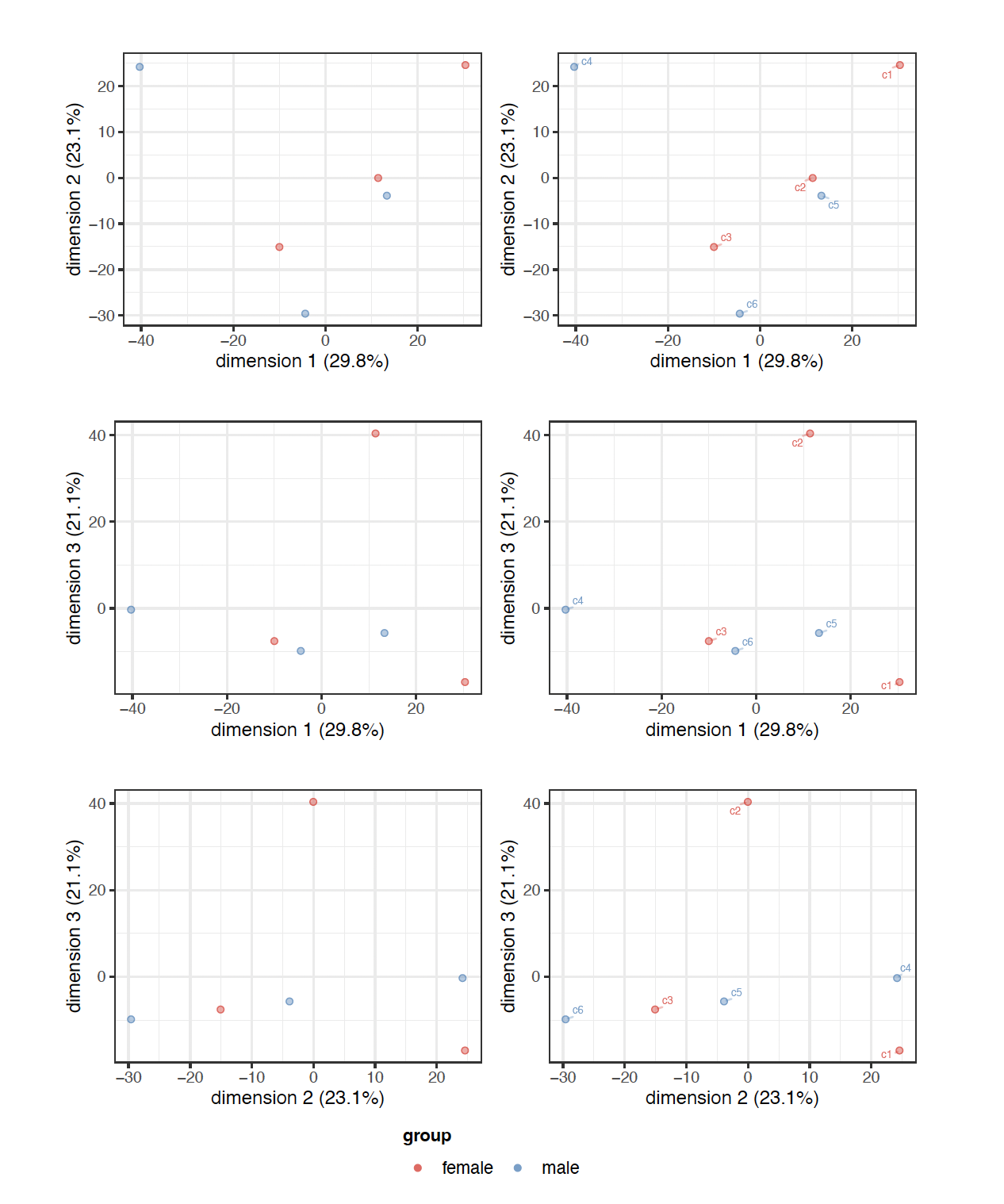
**

**Fig. S7: Principal Component Analysis (male vs female).** The first 3 principal components compared visually (1 vs 2, 1 vs 3, 2 vs 3) on the rows. Left- and right-side panels on each row represent the same figure without and with sample labels. The principal components are shown on the axis labels together with their respective percentage of variance explained. The pcaMethods R package is used here to perform the Probabilistic PCA (PPCA).

**S8: Abundance distribution of complement pathway proteins relative to the global proteome**


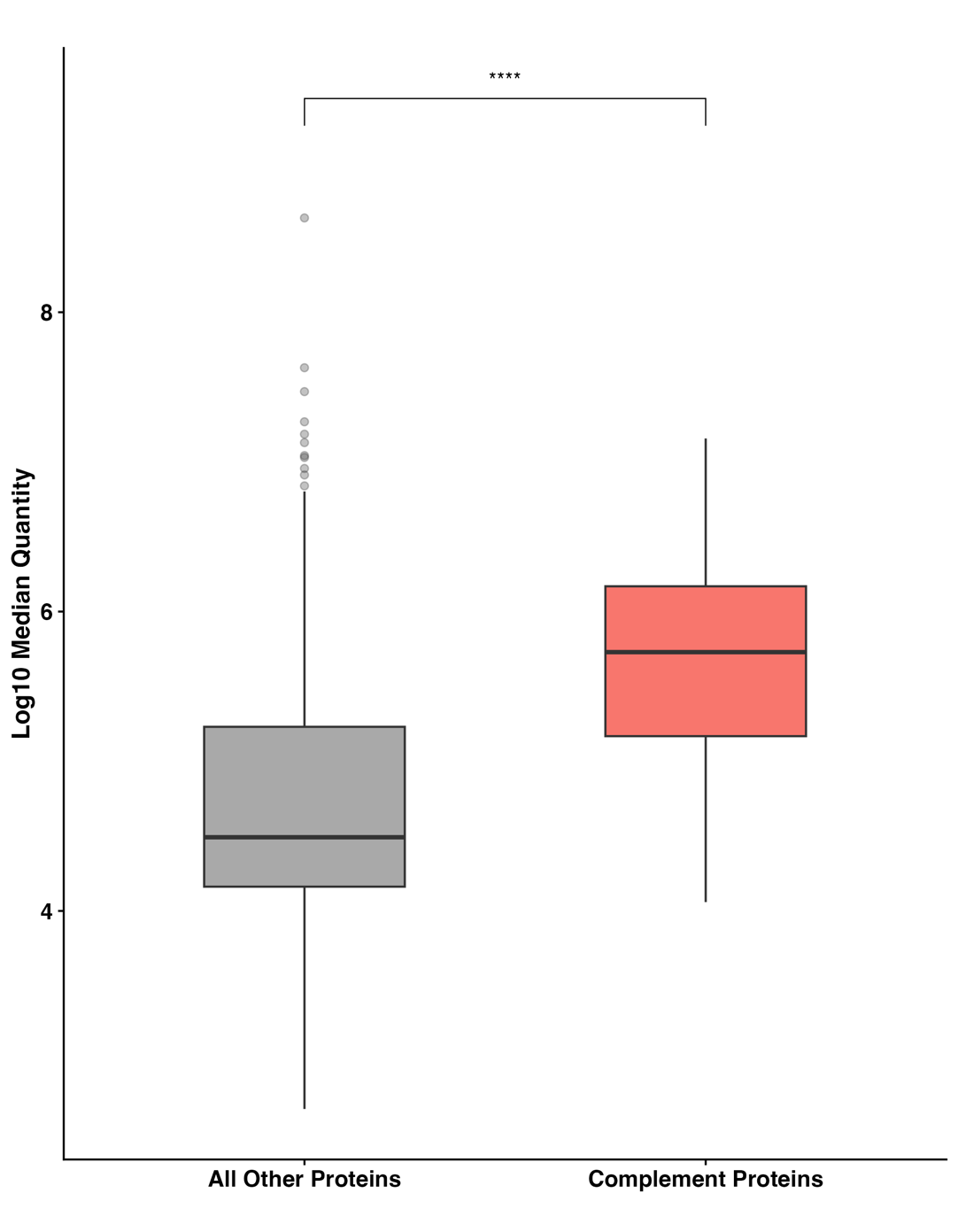


**Fig. S8. Abundance distribution of complement pathway proteins relative to the global proteome.** Boxplot comparing the log_10_-transformed median abundance of detected complement proteins (n = 24) against the remainder of the detected proteome across both sexes (n = 303). Complement components exhibited a significantly higher abundance profile (Wilcoxon rank-sum test, p = 6.80 x 10^-7^), with a median log_10_ abundance of 5.73 (IQR: 5.17–6.17) compared to a proteome-wide median of 4.49 (IQR: 4.16–5.23). The center line represents the median, the box denotes the interquartile range (IQR). These data support the consistently high representation of the complement system within the ERB proteome, with the median complement protein being approximately 17-fold more abundant than the proteome average.

**Figure S9. Differential detection histogram (female vs male).**

**
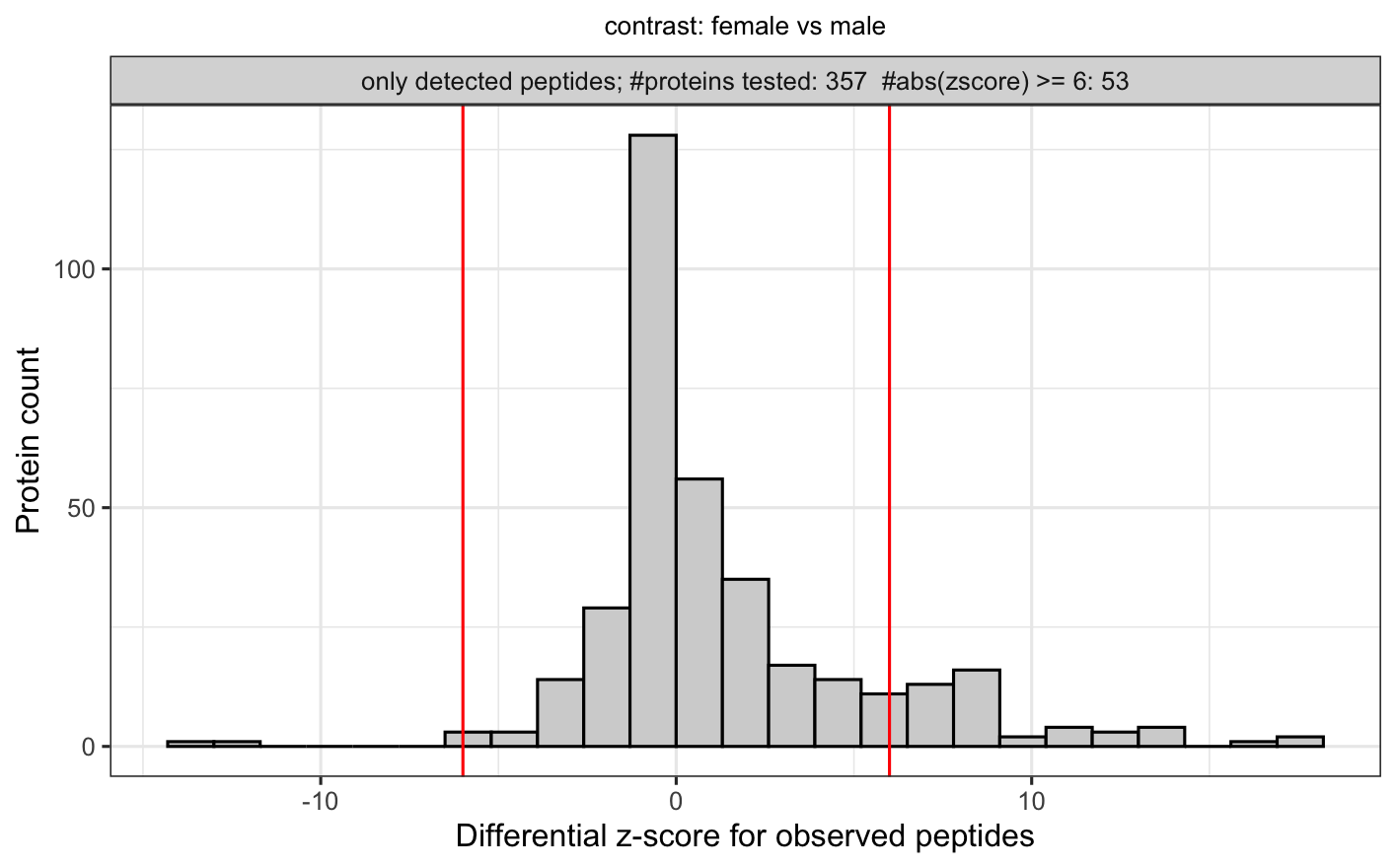
**

**Fig. S9. Differential detection histogram (female vs male).** Differential detection shows a summary of proteins with extreme differences in observed peptides. A simple metric to complement results from DAA/DEA, which is the main result, especially for proteins that lack data to perform differential expression-based analyses. The above figure shows the distribution of these scores (positive z-scores = higher in males) with thresholds at 6 standard deviations. Both the z-scores and the counts these are based upon are available in the statistical result supplementary Excel table (ST5)

**
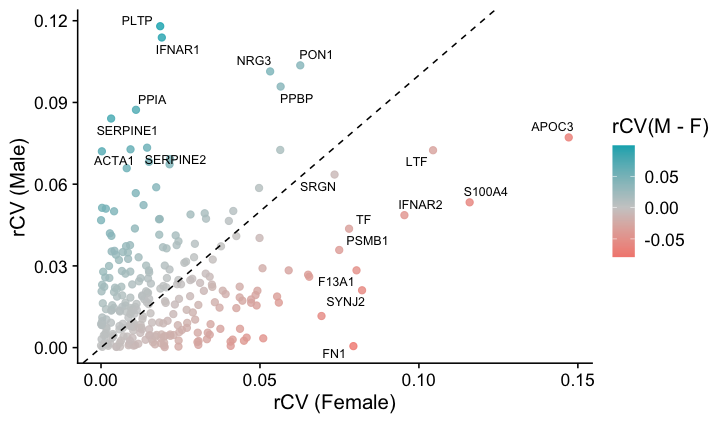
Figure S10. Coefficient of variation (rCV) comparison between sexes for proteins quantified in both male and female ERB serum.**

**Fig. S10.** **Coefficient of variation (rCV) comparison between sexes for proteins quantified in both male and female ERB serum.** Scatterplot of relative coefficients of variation (rCV, defined as MAD/median abundance) for proteins detected in both sexes. Points represent individual proteins; diagonal dashed line indicates equal variability between sexes. Color scale reflects difference in rCV between males (teal) and females (pink). The ten most variable proteins within each sex are annotated using their human ortholog gene name symbol. The use of rCV ensured that variability estimates were standardized by median abundance, minimizing bias from proteins differing primarily in absolute abundance.

Among the most variable proteins, apolipoprotein C-III (APOC3) emerged as an important outlier having qualified as a highly variable protein in both sexes (rCV = 0.15 in females, rCV = 0.08 in males) suggesting a shared source of individual heterogeneity. In contrast, other proteins like phospholipid transfer protein (PLTP), paraoxonase 1 (PON1), interferon alpha/beta receptor 1 (IFNAR1), showed sex-dependent differences that were more pronounced in male ERBs than females. We found that interferon alpha/beta receptor 2 (IFNAR2; rCV = 0.09 in females), the high affinity subunit of the IFNAR complex, showed more variation within females than males. These proteins may reflect sex-specific heterogeneity in regulation or post-transcriptional control and warrant further investigations.

By comparison, several proteins showed exceptional within-sex stability. In females, complement C7 (C7), apolipoprotein A I (APOA1), and transforming growth factor-beta-induced protein ig-h3 (TGFBI) exhibited very low rCV values (<0.001), indicating highly consistently expression across individuals. In males, proteins such as vitamin D-binding protein (GC), extracellular matrix protein 1 (ECM1), and angiotensinogen (AGT) were similarly stable (rCV < 0.003). Full results from both variable and stable proteins in each sex are available in the supplemental materials (S6).

**S11. Methods for protein variability analysis**

To evaluate within-sex variability in protein abundances, we first restricted the dataset to proteins that were quantified in all three biological replicates of both sexes. For each protein within each sex, we calculated the median absolute deviation (MAD) and the median abundance. From these values we derived the relative coefficient of variation (rCV = MAD/median) as a robust measure of dispersion that is less sensitive to outliers than the standard deviation. For comparison across sexes, we plotted rCV values and labeled the top 10 most variable proteins within each sex. The full list of most variable and most stable proteins per sex can be found in the supplemental materials (S6).
